# Adipose Tissue and Mesenchymal Stem Cells: State of the Art and Lipogems® Technology Development

**DOI:** 10.1007/s40778-016-0053-5

**Published:** 2016-07-13

**Authors:** Carlo Tremolada, Valeria Colombo, Carlo Ventura

**Affiliations:** 1 Image Institute, Milan, Italy; 2 Stem Wave Institute for Tissue Healing (SWITH)—Ettore Sansavini Health Science Foundation, Lugo, Ravenna, Italy

**Keywords:** Mesenchymal stem cell, Lipogems®, Adipose tissue, Regenerative medicine, Adipose tissue-derived mesenchymal stem cell, Adult stem cells

## Abstract

In the past few years, interest in adipose tissue as an ideal source of mesenchymal stem cells (MSCs) has increased. These cells are multipotent and may differentiate in vitro into several cellular lineages, such as adipocytes, chondrocytes, osteoblasts, and myoblasts. In addition, they secrete many bioactive molecules and thus are considered “mini-drugstores.” MSCs are being used increasingly for many clinical applications, such as orthopedic, plastic, and reconstructive surgery. Adipose-derived MSCs are routinely obtained enzymatically from fat lipoaspirate as SVF and/or may undergo prolonged ex vivo expansion, with significant senescence and a decrease in multipotency, leading to unsatisfactory clinical results. Moreover, these techniques are hampered by complex regulatory issues. Therefore, an innovative technique (Lipogems®; Lipogems International SpA, Milan, Italy) was developed to obtain microfragmented adipose tissue with an intact stromal vascular niche and MSCs with a high regenerative capacity. The Lipogems® technology, patented in 2010 and clinically available since 2013, is an easy-to-use system designed to harvest, process, and inject refined fat tissue and is characterized by optimal handling ability and a great regenerative potential based on adipose-derived MSCs. In this novel technology, the adipose tissue is washed, emulsified, and rinsed and adipose cluster dimensions gradually are reduced to about 0.3 to 0.8 mm. In the resulting Lipogems® product, pericytes are retained within an intact stromal vascular niche and are ready to interact with the recipient tissue after transplantation, thereby becoming MSCs and starting the regenerative process. Lipogems® has been used in more than 7000 patients worldwide in aesthetic medicine and surgery, as well as in orthopedic and general surgery, with remarkable and promising results and seemingly no drawbacks. Now, several clinical trials are under way to support the initial encouraging outcomes. Lipogems® technology is emerging as a valid intraoperative system to obtain an optimal final product that may be used immediately for regenerative purposes.

## Introduction

More than a century has passed since the first definition of stem cells as ancestral cells of the germ line [1], and since then, a significant number of studies and discoveries concerning their potential and application in regenerative medicine and surgery have been published. In particular, more than 40,000 articles may be found on Medline by searching for human adult mesenchymal stem cells (MSCs) derived from bone marrow, dental pulp, fetal membrane, and term placenta. In vitro and in vivo experimental studies have demonstrated that human MSCs may differentiate in vitro into several cell lineages, such as osteoblasts, chondrocytes, myocytes, and adipocytes [2].

In the past 15 years, it has been shown that human MSCs also can promote vasculogenesis, the main mechanism involved in tissue repair effectiveness, cardiovascular differentiation, and myocardial repair [3], and have improved islet graft revascularization in diabetic rats, enhancing engraftment success [4]. All the aforementioned discoveries inspired the studies regarding Lipogems® (Lipogems International SpA, Milan, Italy) technology.

Attention increasingly is being focused on MSCs derived from human and animal adipose tissue because of their abundance and ease of access. These multipotent cells can differentiate into mature adipocytes as well as chondrocytes, osteoblasts, myocytes, hepatocytes, neuronal-like and endothelial cells, and other lineages, as suggested by in vitro, ex vivo, and in vivo evidence [5–14], and this potential may be used to regenerate damaged tissues. In addition, MSCs secrete a variety of bioactive molecules that act in a paracrine fashion to prime and sustain angiogenic, antifibrotic, antiapoptotic, and immunomodulatory responses in target tissue [2, 15•].

Adipose-derived MSCs routinely are obtained enzymatically and may undergo prolonged ex vivo expansion, with significant senescence and a decline in multipotency. In addition, the technique is fraught with complex regulatory issues.

This review presents an overview of the knowledge and clinical applications of adult MSCs, highlighting the role Lipogems® technology has played in regenerative medicine so far. The urgent need to find new therapies for chronic immunologic and degenerative diseases prompted many investigators to search for products containing progenitor cells while avoiding the problems and restrictions related to enzymatic manipulation and cell expansion in accordance with good manufacturing practice (GMP) rules [16, 17].

The availability of minimally manipulated products based on adequate MSC content has resulted in shorter procedure times and the ability to apply autologous grafts in a one-step intervention. The Lipogems® technology guarantees both these requirements in an easy-to-use, rapid, and disposable adipose tissue transfer device and represents a very promising approach.

### MSCs, the Medicine of the Future: Sources and Purposes

MSCs can proliferate in vitro and have multipotent differentiation properties. They also are strong immune modulators, inhibiting proinflammatory processes and stimulating anti-inflammatory mechanisms. These features may be exploited to treat specific degenerative and inflammatory diseases in the near future; thus, research into the isolation, manipulation, and expansion of MSCs is increasing.

MSCs can be selected in culture from almost any tissue, including dental pulp, periodontal ligament, bone marrow, fetal membranes, and placenta [18, 19]. Recently, they were recognized in vivo as being derived from perivascular cells [20, 21] and hence “injury-specific” cells. Indeed, MSCs detach from vessels and become “medicinal signaling cells” that can receive signals of injury from the environment and then respond by secreting the appropriate molecules [22]. Dental pulp is considered an effective MSC source for orthopedic and maxillofacial reconstructions, because these MSCs can generate mineralized tissue, extracellular matrix, and other connective tissue, such as dentine, dental pulp, and periodontal ligament [23]. Although MSCs clearly may be used to regenerate the dental pulp itself [24, 25] and may also influence the pathogenic pathways of some chronic brain and gut diseases (e.g., Parkinson’s and Alzheimer’s) [26], a treatment based on these cells is not yet available. Indeed, evidence obtained so far has been gathered only by using nonhuman xenotransplants and in vitro models. Bone marrow-derived MSCs also have been studied extensively for their great regeneration capability and their immunosuppressive capacity [27]. Several protocols for cell culture and expansion have been established, and many efforts have been aimed at finding optimal conditions for clinical-scale production of MSCs for cellular and gene therapy for inherited and acquired diseases [28].

Although many ex vivo and in vitro protocols have been proposed, only a few studies have been done on the clinical applications of MSCs. The benefit of bone marrow MSC therapy in improving liver fibrosis was described in 11 patients with alcoholic cirrhosis [29]. Another study in two patients with compensated cirrhosis investigated whether intraportal injection of autologous bone marrow-derived MSCs combined with pioglitazone could stop or reduce liver fibrosis. Although the results were encouraging, studies with more patients are needed to prove the effectiveness of this clinical application [30, 31].

In 2010, Mazzini and Ferrero [32] began the first clinical trial to use bone marrow-derived MSCs to treat amyotrophic lateral sclerosis and obtained promising results regarding the safety and usefulness of this procedure. Moreover, a recent review on the treatment of spinal cord injuries with bone marrow MSC transplantation in 20 patients reported convincing functional improvement [33]. More recently, multiple intramedullary and intradural transplantations of MSCs were shown to achieve more satisfying results versus a single transplant [34]. In 2005, a preliminary clinical trial using adipose-derived MSCs to treat Crohn’s fistula was described, but further studies with larger patient samples are needed to prove the feasibility of this application [35].

All the aforementioned clinical trials were hampered by complex and time-consuming enzymatic manipulation of MSCs, as well as the extra centrifugation step(s) needed to obtain an adequate amount of cells. Because of the need to avoid these cumbersome procedures, the search for newer and better sources and processing strategies became increasingly urgent.

### Fat Tissue Potential: an Ideal MSC Source

The idea that fat tissue is an optimal source of MSCs is supported by the abundance of these cells in this tissue compared with other tissues, such as the widely used bone marrow, and by their easy access [36••, 37–39]. Indeed, 1 in 100 adipose tissue cells is an MSC, compared with 1 in 100,000 bone marrow cells. Moreover, bone marrow harvesting is an invasive and traumatic procedure compared with lipoaspiration and is performed under general anesthesia with a higher risk of viral infection. Finally, it is widely recognized that the viability and differentiation capacity of bone marrow-derived MSCs decrease with increasing donor age [40]. Although other sources, such as dental pulp, umbilical cord matrix (Wharton jelly), and menstrual blood, have been considered, their isolation and the amplification steps involved are time consuming and require careful laboratory manipulation [41–43].

Fat tissue is available in large quantities in most patients and can be harvested easily with a minimally invasive approach (under either local or general anesthesia), offering a highly viable MSC population with optimal differentiation potential independent of the donor’s age. The regenerative potential of adipose tissue-derived MSCs is similar to that reported in other tissues.

In the past few years, several studies focused on the technical improvement and the maximization of the therapeutic effects of the traditional fat transfer and structural fat grafting technique described by Coleman [44, 45]. Tissue engineering in vitro was developed to guarantee an optimal amount of MSCs in the transplanted fat (expansion of the stromal vascular fraction and the adult multipotent elements). However, as stated earlier, the current challenge lies in obtaining tissue that has been manipulated minimally and contains an effective amount of progenitor cells and MSCs, possibly bypassing the long enzymatic manipulation phase and GMP restrictions.

### Adipose Tissue Graft Preparation Methods

New processing technologies, such as the Puregraft (Puregraft, Solana Beach, CA) [46] and Tulip (Tulip Medical Products, San Diego, CA) systems [47], have been developed to obtain ready-to-use, minimally manipulated autologous MSC products. Simple lipoaspiration, gravity separation, Coleman fat centrifugation, and microfat and nanofat techniques are the most cited and used approaches. A recent study compared the classical lipofilling technique with three commercial devices to obtain a fat derivative enriched in MSCs, confirming that a greater amount of MSCs leads to better and more stable results [48]. Several studies showed greater tissue viability and a lower percentage of contaminants in fat tissue washed and filtrated within a closed system [49].

Among these novel achievements, the Lipogems® technology has an emerging role.

Few practical clinical studies have been published on the use of adipose tissue-derived MSCs in humans. Recently, some authors applied MSCs derived from the buccal fat pad combined with iliac crest bone grafting to reconstruct an atrophic alveolar ridge and reported increased new bone formation [50]. More clinical studies are needed to elucidate the real clinical effects of MSCs.

### Lipogems® Technology and Rationale

Lipogems® is a simple system designed to harvest, process, and transfer refined adipose tissue and is associated with great regenerative potential and optimal handling ability. With the help of this new technology, and without enzymes or other additives, fat tissue is microfragmented gently and washed from proinflammatory oil and blood residues. The resulting product contains pericytes retained within an intact stromal vascular niche and is ready to interact with the recipient tissue after transplantation, thereby becoming activated as MSCs.

**Fig. 1:**
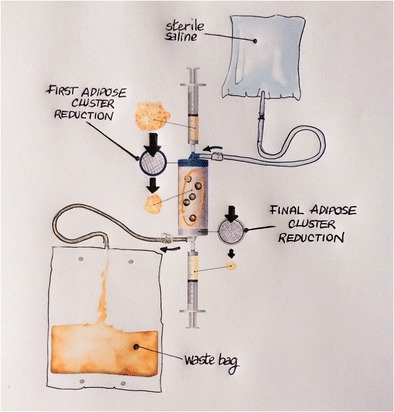
Mechanisms of the Lipogems® device

The first step in this procedure is aspiration of a small quantity of fat tissue from the donor site. With the donor under local anesthesia, a skin incision of a few millimeters is made and Klein solution is injected into the subcutaneous fat tissue of the site. Fat tissue is harvested and processed with the Lipogems® device (Fig. 1), a closed, full-immersion, low-pressure cylindrical system, to obtain fluid and a uniform product containing many pericytes/MSCs. Throughout the procedure, the processed fat is subjected to only slight mechanical forces, with no detrimental effects on the integrity of the stromal vascular niche and or the tissue itself [36••], and is ready for use in the required clinical application [51].

With regard to emerging clinical, scientific, and regulatory requirements, the Lipogems® technology bases its effectiveness on five basic principles. First, Lipogems® preserves viable elements with pericyte identity within an intact stromal vascular niche. It now is evident that cells defined as MSCs exhibit substantial perivascular location and pericyte identity in vivo. Pericytes are structural cells in the external wall of the microvessels and capillaries of the stromal vascular fraction of the adipose tissue. After an injury, such as inflammation or damage to the vascular wall, pericytes detach from the capillaries and gradually convert into activated regenerative MSCs [36••]. Hence, the Lipogems® product allows posttransplant availability of its own pericytes for activation to MSCs where/when required by the recipient tissue. Such pericyte-to-MSC activation entails the release of regenerative factors, causing the transplanted Lipogems® product to act as a “time-release medium” of these factors where they are most needed [36••].

Second, after injection, the MSCs begin to produce a complex and heterogeneous spectrum of bioactive molecules, which are secreted in exosomes and act in a paracrine fashion in the surrounding environment. The exosome content has been demonstrated to be significantly greater in fat tissue processed with Lipogems® than with the enzymatic method [52–57]. The reasons for this finding are as follows: (1) enzymatic treatment of cells digests the extracellular matrix surrounding the cells, possibly affecting cell secretory functions; (2) the digestion also might damage the cells, affecting cell function and viability; (3) the enzymatic method may be too aggressive and might destroy exosomes during processing. The Lipogems® system, in contrast, is relatively gentle, and the reduced tissue clusters maintain cells in a more “native” environment, which may help support cell function, including exosome release and secretion.

Third, Lipogems® can activate the same cascade of biological events, respecting the natural healing process. The product is a minimally manipulated microfragmented adipose tissue that enhances the natural regenerative properties of the receiving tissue [54]. The main structural and morphologic unit, the adipose niche, is maintained after processing and protects the activated MSCs, strengthening their effectiveness in the recipient environment. This is a fundamental difference between Lipogems® and other methods, because preserving the adipose structural niches increases MSC efficacy [55]. In addition, the elimination of fat centrifugation and enzymatic treatment minimizes tissue trauma and preserves cell integrity. The decrease in adipose cluster dimension makes handling and posttransplant engraftment easier because of a more effective and faster graft revascularization.

Fourth, the gentle mechanical method allows a ready-to-use product to be obtained in less than 20 min, compared with the several hours, days, or weeks required for enzymatic digestion of the lipoaspirate, and possibly in vitro cell expansion, resulting in substantial delays in clinical application.

Fifth, Lipogems® is minimally manipulated according to the regulations set forth by the US Food and Drug Administration. Lipogems® qualifies as a “361 HCT/P” (human cells, tissues, and cellular and tissue-based product under section 361 of the Public Health Service Act) because it is (1) autologous, (2) minimally manipulated, (3) intended for homologous use, (4) enzyme-free, (5) not dependent on the metabolic activity of the cells for its primary function, (6) used in the same surgical procedure, and (7) not combined with anything other than saline.

In summary, the Lipogems® technology improves and optimizes the natural properties of adipose tissue. Without the use of enzymes, additives, or separation centrifugation and relying instead on mild mechanical forces, the Lipogems® system yields a microfragmented autologous adipose tissue that acts as a large-scale tool to supply damaged tissues with a regenerative environment. The availability of minimally manipulated products based on adequate MSC content allows for shorter procedure times, avoids regulatory constraints, and enables autologous grafting in a one-step intervention. Lipogems® technology meets all these requirements in a simple, rapid, and disposable fat tissue transfer device and represents a very promising approach.

### Lipogems® Clinical Applications

Thanks to the optimal size of the clusters, allowing easy injection of the product, Lipogems® has been tested and used safely in various clinical applications [36••]; so far, no infections or major complications have been reported. The very low infection risk is a result of the production of the antimicrobial molecule LL-37. Moreover, Lipogems® has proved to be very effective mainly in clinical regenerative cases, although adequate scientific support from formal clinical trials is needed to confirm these promising results. Nevertheless, a few studies specifically related to the use of Lipogems® in clinical practice have already been published; however, because of the lack of literature currently available, it is difficult to compare this technique with other methods [36••].

Although results have been published regarding the use of traditionally harvested fat tissue in vocal palsy treatment, complications have been reported, especially in relation to overinjection of fat tissue [58, 59]. In contrast, Lipogems® recently was tested in three patients with vocal cord palsy; during 12 months of follow-up, all the patients had gradual but consistent improvement in their voice and no reported complications. Although these initial results are very encouraging, more cases are required to confirm the regenerative functional potential of adipose tissue-derived MSCs [60].

As this technique has become more widespread, applications for its use have increased, mainly in general, orthopedic, plastic reconstructive and aesthetic, and oral–maxillofacial surgery.

#### General Surgery

In the field of general surgery, Lipogems® has been used as a valid approach for treating fecal incontinence. Cestaro et al. [61] reported great improvement in incontinence score, thickness of the internal anal sphincter, and resting pressure in three patients treated with Lipogems® injection into the anal sphincter. The same encouraging results were obtained by other researchers [62, 63].

In oncology, Lipogems® is useful and powerful when injected in atrophied tissue after radiotherapy. Promising results in terms of restoration of skin elasticity and thickness have been reported in patients who had head and neck surgery and breast reconstruction. Encouraging results also have been found in burn patients (unpublished data).

#### Orthopedic Surgery

Patients who have orthopedic surgery for joint degenerative and inflammatory diseases may benefit from intra-articular injection of Lipogems®. Its regenerative potential in musculoskeletal diseases already has been reported; however, more extensive clinical trials must be performed to confirm the initial results [64].

Based on clinical results from more than 800 patients worldwide (obtained from many European and American colleagues), the intra-articular injection of Lipogems® to treat knee, ankle, hip, and shoulder osteoarthritis resulted in a surprising improvement in symptoms, with 100 % safety of the procedure. After Lipogems® injection (2–12 mL depending on the joint), patients generally reported a striking improvement in symptoms, knee function, and pain. Some patients who were candidates for surgery/prosthesis no longer needed it because of the complete or substantial resolution of their symptoms. In addition, single-case reports have demonstrated that intra-articular injection of Lipogems® in patients with osteoarthritis and nonresponsive knee pain in association with meniscal damage seems to improve joint functionality [65], and recently, intra-articular injection of Lipogems® improved knee function in a patient with a posttraumatic lesion of the cartilage [66].

The orthopedic use of Lipogems® also has been tested in injured ligaments or tendons, in meniscal lesions, around surgical wounds, and inside and around osteotomy gaps. A recent publication shows that Lipogems® may enhance in vitro proliferation of human tendon stem cells and induce greater expression of vascular endothelial growth factor, which is fundamental for neovascularization during the healing process [67].

A recent case report described the efficacy of Lipogems® injection combined with disc decompression in a patient with recurrent low back pain, with promising results [68].

#### Plastic Reconstructive and Aesthetic Surgery

In the area of plastic reconstructive surgery, Lipogems® helps the healing of chronic ulceration of the lower legs and feet, especially in diabetic patients, with optimal results: in our experience, 76 % of patients had complete healing in less than 6 months, with no evidence of recurrence. Only the vasculogenic properties of the MSCs can explain this success [69].

Aesthetic surgery is a growing field of application [70] for Lipogems®, used alone or in association with traditional surgical techniques such as facelift, blepharoplasty, and breast augmentation. In the latter case, Lipogems® accelerates wound healing and improves skin texture. Optimal results have been obtained in treating the tear trough and moderate puffiness of the lower eyelid with Lipogems® injected into the periorbital area, deep to the orbicularis oculi muscle. After surgical intervention, patients report no pain, swelling, or bruising and generally are satisfied. This treatment often is extended to full-face biorestoration aimed at defining facial contour, providing tone, brightness, and uniformity. Unlike simply washed lipoaspirate or Coleman’s fat, Lipogems® shows no evidence of increasing tissue volume.

#### Oral–Maxillofacial Surgery

A recent publication describes the use of Lipogems® during orthognathic surgery in 120 patients who underwent a double-jaw intervention. Lipogems® was injected in multiple tissue planes and tunnels where soft tissue had to be restored (midface and mandibular contours, neck, lips, chin profile). The results were compared with those from several patients treated with traditional lipofilling. Only two patients treated with the Lipogems® technique showed no adjunctive effect or improvement. All the other patients had enhanced facial morphology and skin texture and much less postoperative swelling, resulting in faster recovery [71].

Lipogems® seems to enhance healing, osteointegration, and the stability of implants in the bone, especially in patients with bone atrophy and healing difficulty [72].

### Lipogems, MSCs, and Future Perspectives

Autologous fat tissue transfer is a well-established method with several clinical applications. The Lipogems® technology is thought to be an improvement over traditional grafting techniques [36••].

Stromal vascular fraction is shown to be better preserved in Lipogems® than in native lipoaspirate [36••]. Moreover, immunohistochemical and flow cytometry analyses revealed that Lipogems® contains viable pericytes and MSCs. When the Lipogems® product is placed in culture, these cells are released into the medium and begin to expand after 2 to 3 days, reaching confluence in the tissue culture flasks in 7 to 10 days [36••].

The most important and attractive feature of Lipogems® technology, and of MSCs in general, is cellular multipotency and the capacity to induce repair and regeneration naturally in certain types of tissue. A recently published study compared the effects of placing Lipogems®, native lipoaspirate, centrifuged Coleman’s fat, and Puregraft in chondrogenic media and found that Lipogems® is the best source for creating new cartilage tissue and that regardless of the processing method, the fat tissue represented an ideal scaffold to induce cartilage repair and formation compared with isolated MSCs [73].

Recently, it was demonstrated that exposure of Lipogems®-derived MSCs to properly conveyed radioelectric fields could optimize stem cell expression of multipotency and lineage commitment at a remarkably higher degree than enzymatically dissociated MSCs obtained from the same donors [74, 75].

The regenerative potential of fat tissue in orthopedic and musculoskeletal fields is attracting the attention of an increasing number of researchers, prompting their contribution to adipose derived-stem cell research and clinical application. Recently, Del Papa et al. [76] described the use of adipose tissue-derived cell fraction in successfully treating digital ulcers unresponsive to conventional therapy in 15 patients with systemic sclerosis. Promising results were also obtained by applying this technique to the perioral area and lips in the same group of patients [77]. It is unclear, however, which cellular elements were responsible for the improvement observed in skin and mucosal texture, as well as for the ulcers’ healing, because the authors used the traditional Coleman fat grafting technique with no mechanical or enzymatic manipulation of lipoaspirate-derived adipose tissue.

Conversely, other authors found advanced regenerative methods to be more complete and efficient. A recent review of knee cartilage restoration outlined two approaches: direct intra-articular injection of an adequate population of MSCs and implantation of engineered constructs of MSC-seeded scaffolds [78].

Within this context, the Lipogems® technology fulfills the requirement to overcome the current limitations related to in vitro fat manipulation, making MSCs easily available within their natural three-dimensional scaffold so that they can direct the regeneration of damaged tissues by exosome-mediated signaling. However, it must be stressed that the International Federation for Adipose Therapeutics and Science (IFATS) and International Society for Cellular Therapy (ISCT) recently established clear definitions of stromal vascular fraction and adipose-derived MSCs to better manage future trials and to enable multicenter comparative studies [79–81].

## Conclusions

Adipose tissue is the ideal source for extracting MSCs because (i) it can be accessed and harvested easily via a minimally invasive surgical procedure, (ii) it may be found in large quantities in most people, and (iii) it guarantees an adequate amount of stem cells with good viability and age-related differentiating potential. The Lipogems® technology improves and optimizes the natural properties of adipose tissue. Without using enzymes, additives, or separation centrifugations and relying instead on the use of mild mechanical forces, the Lipogems® system yields a microfragmented autologous adipose tissue that acts as a large-scale tool to supply damaged tissues with a regenerative environment. The availability of minimally manipulated products based on adequate MSC content shortens procedure times, avoids regulatory constraints, and allows autologous grafting in a one-step intervention. The Lipogems® technology meets these requirements in an easy-to-use, rapid, and disposable fat tissue transfer device and represents a promising approach to be tested in additional multicenter studies.

## References

[1] Wilson EB (1898). The cell in development and inheritance.

[2] Caplan AI (2007). Adult mesenchymal stem cells for tissue engineering versus regenerative medicine. J Cell Physiol.

[3] Ventura C, Cantoni S, Bianchi F, Lionetti V, Cavallini C, Scarlata I (2007). Hyaluronan mixed esters of butyric and retinoic acid drive cardiac and endotelial fate in term placenta human mesenchymal stem cells and enhance cardiac repair in infarcite rat hearts. J Biol Chem.

[4] Cavallari G, Olivi E, Bianchi F, Neri F, Foroni L, Valente S (2012). Mesenchymal stem cells and islet cotransplantation in diabetic rats: improve islet graft revascularization and function by human adipose tissue-derived stem cells preconditioned with natural molecule. Cell Transplant.

[5] Canaider S, Maioli M, Facchin F (2014). Human Stem Cell exposure to developmental stage zebrafish extracts: a novel strategy for turning stemness and senescence patterning. CellR4.

[6] Cao Y, Sun Z, Liao L, Meng Y, Han Q, Zhao RC (2005). Human adipose tissue-derived stem cells differentiate into endothelial cells in vitro and improve postnatal neovascularization in vivo. Biochem Biophys Res Commun.

[7] Fraser JK, Schreiber R, Strem B, Zhu M, Alonso Z, Wulur I, et al. Plasticity of human adipose stem cells towards endothelial cells and cardiomyocytes. Nat Clin Pract Cardiovasc Med. 2006;3(Suppl1):S33–7. 10.1038/ncpcardio0444 16501628

[8] Sen A, Lea-Currie YR, Suijkowska D, Franklin DM, Wilkison WO, Halvorsen YD (2001). Adipogenic potential of human adipose derived stromal cells from multiple donors is heterogeneous. J Cell Biochem.

[9] Zuk PA, Zhu M, Ashjian P, De Ugarte DA, Huang JI, Mizuno H (2002). Human adipose tissue is a source of multipotent stem cells. Mol Biol Cell.

[10] Zuk PA, Zhu M, Mizuno H, Huang J, Futrell JW, Katz AJ (2001). Multilineage cells from human adipose tissue: implications for cell-based therapies. Tissue Eng.

[11] Erickson GR, Gimble JM, Franklin DM, Rice HE, Awad H, Guilak F (2002). Chondrogenic potential of adipose tissue-derived stromal cells in vitro and in vivo. Biochem Biophys Res Commun.

[12] Halvorsen YC, Wilkison WO, Gimble JM (2000). Adipose-derived stromal cells—their utility and potential in bone formation. Int J Obes Relat Metab Disord.

[13] Halvorsen YD, Franklin D, Bond AL, Hitt DC, Auchter C, Boskey AL (2001). Extracellular matrix mineralization and osteoblast gene expression by human adipose tissue-derived stromal cells. Tissue Eng.

[14] Huang JI, Beanes SR, Zhu M, Lorenz HP, Hedrick MH, Benhaim P (2002). Rat extramedullary adipose tissue as a source of osteochondrogenic progenitor cells. Plast Reconstr Surg.

[15.•] Caplan AI, Dennis JE (2006). Mesenchymal stem cells as trophic mediators. J Cell Biochem.

[16] Roseti L, Serra M, Tigani D, Brognara I, Lorpiore A, Bassi A (2008). Cell manipulation in autologous chondrocyte implantation: from research to clean room. Chir Organi Mov.

[17] Riis S, Zachar V, Boucher S, Vemuri MC, Pennisi CP, Fink T. Critical steps in the isolation and expansion of adipose-derived stem cells for translational therapy. Expert Rev Mol Med. 2015;17:e11. 10.1017/erm.2015.10 26052798

[18] Caplan A (1991). Mesenchymal Stem Cells. J Orthopedic Res.

[19] Caplan A, Bruder SP (2001). Mesenchymal stem cells: building blocks for molecular medicine in the 21st century. Trends Mol Med.

[20] Caplan A (2008). All MSCs are pericytes?. Cell Stem Cell.

[21] Crisan M, Yap S, Casteilla L, Chen CW, Corselli M, Soon Park T (2008). A perivascular origin for mesenchymal stem cells in multiple human organs. Cell Stem Cell.

[22] Da Silva Meirelles L, Caplan A, Beyer Nardi N (2008). In search of the in vivo identity of mesenchymal stem cells. Stem Cells.

[23] Ledesma-Martinez E, Mendoza-Nunez VM, Santiago-Osorio E. Mesenchymal stem cells derived from dental pulp: a review. Stem cells Int. 2016;2016:4709572. 10.1155/2016/4709572 PMC4686712 26779263

[24] Kuang R, Zhang Z, Jin X et al. Nanofibrous spongy microspheres for the delivery of hypoxia-primed human dental pulp stem cells to regenerate vascularized dental pulp. Acta Biomater. 2016;33:225–234. 10.1016/j.actbio.2016.01.032 PMC5975264 26826529

[25] Dhillon K, Kaushik M, Sharma R. Regenerative endodontics—creating new horizons. J Biomed Mater Res B Appl Biomat. 2016;104(4):676–85. 10.1002/jbm.b.33587 26699211

[26] Foldes A, Kadar K, Keremi B et al. Mesenchymal stem cells of dental origin-their potential for anti-inflammatory and regenerative actions in brain and gut damage. Curr Neuropharmacol. 2016 (in press). 10.2174/1570159X14666160121115210 PMC5333580 26791480

[27] Solemani M, Nadri S (2009). A protocol for isolation and culture of mesenchymal stem cells from mouse bone marrow. Nat Protoc.

[28] Tonti GA, Mannello F (2008). From bone marrow to therapeutic applications: different behavior and genetic/epigenetic stability during mesenchymal stem cells expansion in autologous and foetal bovine sera?. Int J Dev Biol.

[29] Jang YO, Kim YJ, Baik SK (2014). Histological improvement following administration of autologous bone marrow-derived mesenchymal stem cells for alcoholic cirrhosis: a pilot study. Liver Int.

[30] Vosough M, Moossavi S, Mardpour S (2016). Repeated intraportal injection of mesenchymal stem cells in combination with pioglitazone in patients with compensated cirrhosis: a clinical report of two cases. Arch Iran Med.

[31] Kharaziha P, Hellström PM, Noorinayer B, Farzaneh F, Aghajani K, Jafari F (2009). Improvement of liver function in liver cirrhosis patients after autologous mesenchymal stem cell injection: a phase I-II clinical trial. Eur J Gastroenterol Hepatol.

[32] Mazzini L, Ferrero I (2010). Mesenchymal stem cell transplantation in amyotrophic lateral sclerosis: a phase I clinical trial. Exp Neurol.

[33] Jiang PC, Xiong WP (2013). A clinical trial report of autologous bone marrow-derived mesenchymal stem cell transplantation in patients with spinal cord injury. Exp Ther Med.

[34] Oh SK, Choi KH, Yoo JY, Kim DY, Kim SJ, Jeon SR (2016). A phase III clinical trial showing limited efficacy of autologous mesenchymal stem cell therapy for spinal cord injury. Neurosurgery.

[35] García-Olmo D, García-Arranz M, Herreros D, Pascual I, Peiro C, Rodríguez-Montes JA (2005). A phase I clinical trial of the treatment of Crohn’s fistula by adipose mesenchymal stem cell transplantation. Dis Colon Rectum.

[36.••] Bianchi F, Maioli M, Leonardi E, Olivi E, Pasquinelli G, Valente S, et al. A new non enzymatic method and device to obtain a fat tissue derivative highly enriched in pericyte-like elements by mild mechanical forces from human lipoaspirates. Cell Transpl. 2013;22:2063–77. **This article highlights the basics of Lipogems technology, detailing how to use the device and the properties of the final product**. 10.3727/096368912X657855 23051701

[37] Von Heinburg D, Hemmrich K, Haydarlioglu S (2004). Comparison of viable cell yield from excised versus aspirated adipose tissue. Cells Tissues Organs.

[38] Zhao Y, Betzler C, Popp F et al. Fair or foul: time for standard protocols for potential application of adipose-derived stem cells? Stem Cell Res Ther. 2014;4(7).

[39] Strem BM, Hicok KC, Zhu M (2005). Multipotential differentiation of adipose tissue-derived stem cells. Keio J Med.

[40] Stolzing A, Jones E, McGonegle D (2008). Age-related changes in human bone marrow-derived mesenchymal stem cells: consequences for cell therapies. Mech Ageing Dev.

[41] Vangsness CT, Sternberg H, Harris L (2015). Umbilical cord tissue offers the greatest number of harvestable mesenchymal stem cells for research and clinical application: a literature review of different harvest sites. Arthroscopy.

[42] Ren H, Sang Y, Zhang F, Liu Z, Qi N, Chen Y. Comparative analysis of human mesenchymal stem cells from umbilical cord, dental pulp, and menstrual blood as sources for cell therapy. Stem Cells Int. 2016;2016:13. doi:10.1155/2016/3516574 10.1155/2016/3516574 PMC4736971 26880954

[43] Jeon YJ, Kim J, Cho JH, Chung HM, Chae JI (2016). Comparative analysis of human mesenchymal stem cells derived from bone marrow, placenta, and adipose tissue as sources of cell therapy. J Cell Biochem.

[44] Coleman SR (2006). Structural fat grafting: more than a permanent filler. Plast Reconstr Surg.

[45] Coleman SR (1998). Structural fat grafting. Aesth Surg J.

[46] Mestak O, Sukop A, Hsueh YS (2014). Centrifugation versus PureGraft for fat grafting to the breast after breast-conserving therapy. World J Surg Oncol.

[47] Alexander RW (2011). Autologous fat grafts as mesenchymal stromal stem cell source for use in prolotherapy: a simple technique to acquire lipoaspirants. J Prolotherapy.

[48] Domenis R, Lazzaro L, Calabrese S, Mangoni D, Gallelli A, Bourkoula E (2015). Adipose tissue derived stem cells: in vitro and in vivo analysis of a standard and three commercially available cell-assisted lipotransfer techniques. Stem Cell Res Ther.

[49] Zhu M, Cohen SR, Hicok KC, Shanahan RK, Strem BM, Yu JC (2013). Comparison of three different fat graft preparation methods: gravity separation, centrifugation, and simultaneous washing with filtration in a closed system. Plast Reconstr Surg.

[50] Khojasteh A, Sadeghi N. Application of buccal fat pad-derived stem cells in combination with autogenous iliac bone graft in the treatment of maxillomandibular atrophy: a preliminary human study. Int J Oralmaxillofac Surg. 2016;45(7):864–871. 10.1016/j.ijom.2016.01.003 26846793

[51] Tremolada C. Lipogems International Spa. US patent US9044547 B2, Jun 2015

[52] Garcia-Contreras M, Messaggio F, Jimenez O, Mendez A. Differences in exosome content of human adipose tissue processed by non-enzymatic and enzymatic method. CellR4. 2014;3(1).

[53] Oberbauer E, Steffenhagen C et al. Enzymatic and non-enzymatic isolation systems for adipose tissue-derived cells: current state of the art. Cell Regenerat. 2015; 4(7). 10.1186/s13619-015-0020-0 PMC4591586 26435835

[54] Carelli S, Messaggio F, Canazza A (2015). Characteristics and properties of mesenchymal stem cells derived from microfragmented adipose tissue. Cell Transplant.

[55] Tonnard P, Verpaele A, Peeters G (2012). Nanofat grafting: basic research and clinical applications. Plast Reconstr Surg.

[56] Yu B, Zhang X (2014). Li X exosomes derived from mesenchymal stem cells. Int J Mol Sci.

[57] Rani S, Ryan AE, Griffin MD, Ritter T (2015). Mesenchymal stem cell-derived extracellular vesicles: toward cell-free therapeutic applications. Mol Ther.

[58] Sanderson JD, Simpson CB (2009). Laryngeal complications after lipoinjection for vocal cord augmentation. Laryngoscope.

[59] Young VN, Wijewickrama RC, Pizzuto MA, Rosen CA (2012). An unusual complication of vocal fold lipoinjection: case report and review of the literature. Arch Otolaryngol Head Neck Surg.

[60] Saibene AM, Pipolo C, Lorusso R, Portaleone SM, Felisati G (2015). Transnasal endoscopic microfractured fat injection in glottic insufficiency. B-ENT.

[61] Cestaro G, De Rosa M, Massa S, Amato B, Gentile M (2015). Intersphincteric anal lipofilling with micro-fragmented fat tissue for the treatment of faecal incontinence: preliminary results of three patients. Wideochir Inne Tech Maloinwazyjne.

[62] Giori A, Tremolada C, Vailati R, Navone SE, Marfia G, Caplan AI (2015). Recovery of function in anal incontinence after micro-fragmented fat graft (Lipogems®) injection: two years follow up of the first 5 cases. CellR4.

[63] Testa A, Verdi A, Termini L (2015). New frontiers of the treatment of perianal fistulas: the autologous transplantation of stem cells adult multipotent cells derived from human adipose tissue. 6th National Congress of the Italian Society of Colorectal Surgery Patients First: Quality of Care, Management, Multidisciplinary Approach, Treviso, Treviso, 2015. Tech Coloproctol.

[64] Tremolada C, Beltrami G, Magri A, Bianchi F, Ventura C, Di Vito C (2014). Adipose mesenchymal stem cells and “regenerative adipose tissue graft” (Lipogems®)for musculoskeletal re generation. Eur J Muscoloskeletal Dis.

[65] Striano RD, Chen H, Bilbool N, Azatullah K, Hilado J, Horan K. Non-responsive knee pain with osteoarthritis and concurrent meniscal disease treated with autologous micro-fragmented adipose tissue under continuous ultrasound guidance. CellR4. 2015; 3(5).

[66] Franceschini M, Castellaneta C, Mineo G. Injection of autologous micro-fragmented adipose tissue for the treatment of post traumatic degenerative lesion of knee cartilage: a case report. CellR4. 2016;4(1).

[67] Randelli P, Menon A et al. Lipogems product treatment increases the proliferation rate of human tendon stem cells withouth affecting their stemness and differentiation capability. Stem Cells Int. 2016;4373410. doi:10.1155/2016/4373410 10.1155/2016/4373410 PMC4736573 27057170

[68] Grossi P, Giarratana S, Cernei S, Grossi S, Doniselli FM. Low back pain treated with disc decompression and autologous micro-fragmented adipose tissue: a case report. CellR4. 2016;4(1).

[69] Bianchi F, Olivi E, Baldassarre M, Giannone FA, Laggetta M, Valente S (2014). Lipogems®, a new modality off at tissue handling to enhance tissue repair in chronic hind limb ischemia. CellR4.

[70] Tremolada C, Palmieri G, Ricordi C (2010). Adipose transplantation and stem cells. plastic surgery meets regenerative medicine. Cell Transplant.

[71] Raffaini M, Tremolada C. Micro fractured and purified adipose tissue graft (Lipogems®) can improve the orthognatic surgery outcomes both aesthetically and in postoperative healing. CellR4. 2014; 2(4).

[72] Benzi R, Marfia G, Bosetti M, Beltrami G, Magri AS, Versari S, Tremolada C. Microfractured lipoaspirate may help oral bone and soft tissue re generation: a case report. CellR4. 2015; 3(3).

[73] Bosetti M et al. Human lipoaspirate as autologous injectable active scaffold for one-step repair of cartilage defects. Cell Transpl. 2015;25(6):1043–1056. 10.3727/096368915X689514 26395761

[74] Maioli M, Rinaldi S, Santaniello S, Castagna A, Pigliaru G, Delitala A (2014). Radioelectric asymmetric conveyed fields and human adipose-derived stem cells obtained with a nonenzymatic method and device: a novel approach to multipotency. Cell Transpl.

[75] Ventura C, Bianchi F, Cavallini C et al. The use of physical energy for tissue healing. Eur Heart J Suppl. 2015;17(A).

[76] Del Papa N, Di Luca G, Sambataro D, Zaccara E, Maglione W, Gabrielli A (2015). Regional implantation of autologous adipose tissue-derived cells induces a prompt healing of long-lasting indolent digital ulcers in patients with sistemi sclerosis. Cell Transplant.

[77] Del Papa N, Caviggioli F, Sambataro D, Zaccara E, Vinci V, Di Luca G (2015). Autologous fat grafting in the treatment of fibrotic perioral changes in patients with sistemi sclerosis. Cell Transplant.

[78] Wang Y, Yuan M, Guo QY, Lu SB, Peng J (2015). Mesenchymal stem cells for treating articular cartilage defects and osteoarthritis. Cell Transplant.

[79] Bourin P, Bunnel BA, Casteilla L, Dominici M, Katz AJ, March KI (2013). Stromal cells from the adipose tissue derived stromal vascular fraction and culture expanded adipose tissue-derived stromal/stem cells: a joint statement of the International Federation for Adipose Therapeutics and Science (IFATS) and the International Society for Cellular Therapy (ISCT). Cytotherapy.

[80] Magalon J, Daumas A, Veran J, Magalon G, Rossi P, Granel B (2015). Autologous adipose tissue-derived cells: are we talking about adipose derived stem cells, stromal vascular fraction, or Coleman fat grafting?. Cell Transplant.

[81] Zhao Y, Betzler C, Popp F, Bruns C. Fair or foul: time for standard protocols for potential application of adipose-derived stem cells? Stem Cell Res Ther. 2014; 4(7).

